# ENO3 Inhibits Growth and Metastasis of Hepatocellular Carcinoma *via* Wnt/β-Catenin Signaling Pathway

**DOI:** 10.3389/fcell.2021.797102

**Published:** 2021-12-23

**Authors:** Honglei Cui, Danfeng Guo, Xiaodan Zhang, Yaohua Zhu, Zhihui Wang, Yang Jin, Wenzhi Guo, Shuijun Zhang

**Affiliations:** ^1^ Department of Hepatobiliary and Pancreatic Surgery, The First Affiliated Hospital of Zhengzhou University, Zhengzhou, China; ^2^ Henan Research Centre for Organ Transplantation, Zhengzhou, China; ^3^ Henan Key Laboratory for Digestive Organ Transplantation, Zhengzhou, China

**Keywords:** ENO3, hepatocellular carcinoma, proliferation, metastasis, EMT

## Abstract

β-enolase (ENO3) is a metalloenzyme that functions during glycolysis and has been revealed ectopic expression in different cancers. However, the function and underlying modulatory mechanisms of ENO3 in hepatocellular carcinoma (HCC) are still elusive. Here, we discovered that ENO3 was remarkably down-regulated in human HCC tissue in contrast to those in noncancerous tissue. Moreover, low expression of ENO3 was related to the poor prognosis of HCC patients. Overexpression of ENO3 suppressed proliferative, migratory, and invasive abilities of HCC cells both *in vitro* and *in vivo*, whereas knocking down ENO3 led to the opposite effect. In addition, we revealed that ENO3 repressed the epithelial-mesenchymal transition (EMT) process with its biomarker variations. Mechanistic research unveiled that ENO3 suppressed the Wnt/β-catenin signal, which subsequently modulated the transcription of its target genes associated with the proliferation and metastasis capacity of HCC cells. Taken together, our study uncovered that ENO3 acted as a tumor inhibitor in HCC development and implied ENO3 as a promising candidate for HCC treatment.

## Introduction

HCC, the major form of primary hepatic carcinoma, is one of the most common cancer types and ranks fourth in terms of tumor-related mortality worldwide (Llovet et al., 2021). Different risk factors, such as HBV or HCV infections, cirrhosis, autoimmune hepatitis, nonalcoholic fatty liver disease, and alcohol abuse, may facilitate HCC progression (Villanueva, 2019; Rebouissou and Nault, 2020; Llovet et al., 2021). Moreover, many critical molecular alterations have been revealed during HCC, including *TERT*, *TP53*, and *CTNNB1*, etcetera (Villanueva, 2019; Llovet et al., 2021). HCC cells have tremendous proliferation and metastasis capacities and are prone to drug resistance, which mediates their aggressive behavior (Deldar Abad Paskeh et al., 2021). Despite significant progress in clinical management, the outcomes of HCC patients remain poor due to their rapid development and early recurrence (Ge et al., 2020). Therefore, it’s imperative to define the modulatory causal links of HCC and determine new therapeutic targets.

The canonical Wnt/β-catenin signaling pathway is pivotal for the modulation of cell growth, differentiation, and migration (He and Tang, 2020). β-catenin is the core protein of the Wnt signaling cascades encoded by the *CTNNB1* gene (Perugorria et al., 2019; He and Tang, 2020). Without extracellular Wnt ligands, this pathway is not active and cytoplasm-β-catenin is subjected to be phosphorylated by a sizeable multi-protein complex named the “APC/Axin/GSK-3β complex” and afterward degraded by proteasomes (Perugorria et al., 2019). When the signaling path is activated, Wnt receptors will suppress the APC/Axin/GSK-3β complex-mediated β-catenin phosphorylation, causing the cumulation of β-catenin within the cytoplasm. Subsequently, cytoplasmic β-catenin travels to the nucleus and exhibits interactions with TCF/LEF-1 TFs, causing the transcript of Wnt target genes, like *C-myc*, *CyclinD1*, *MMP2*, *MMP7*, and *MMP9*, which finally result in tumorigenesis and metastasis (Zhou et al., 2019; Liu et al., 2021).

In addition, the Wnt/β-catenin signaling pathway can facilitate EMT (He and Tang, 2020), in which tumor cells lose epithelial features and acquire mesenchymal characteristics and afterward obtain invasiveness and stem cell-like phenotype (Aiello and Kang, 2019). The loss of functional E-cadherin and the acquisition of mesenchymal molecules like Vimentin and N-cadherin are considered biomarkers of EMT (Pastushenko and Blanpain, 2019). EMT is a significant event in cancer progression and metastasis that is regulated by a limited number of TFs, like Snail, Twist, and Zeb families (Yu et al., 2021).

Enolase, composed of three subunits (α, β, γ), catalyzes the glycolytic step interconverting 2-phosphoglycerate and phosphoenolpyruvate. The β-subunit is encoded by the *ENO3* gene and mainly expresses in skeletal muscle and liver (Feo et al., 1995). ENO3 plays an important role in both glycogen and cholesterol metabolism. It has been demonstrated that ENO3 deficiency may lead to metabolic myopathies (Tarnopolsky, 2018), and ENO3 speeds up hepatic cholesterol ester cumulation induced via the mediation of cholesteryl ester generation (Jia and Zhai, 2019). Apart from these, ENO3 has also been involved in various tumors, though the results are contradictory. For example, ENO3 is up-regulated in *STK11* mutant pulmonary carcinoma (Park et al., 2019) and colorectal carcinoma (Xiaohang et al., 2020; Zhu et al., 2021), while down-regulated in pancreatic cancer (Tan et al., 2020) and hepatocellular carcinomas (Liu et al., 2019). Interestingly, down-regulation of ENO3 is also found in the activated human hepatic stellate cells, which can induce liver fibrosis and cirrhosis (Zhong et al., 2015). The above studies indicate ENO3 may not only serve as a catalytic enzyme of cell metabolism but also function in tumor development. Although it’s biologically important, the accurate effects of ENO3 on HCC and the corresponding causal links have not yet been explored.

In the present research, we reveal that ENO3 is regulated downward in HCC, and the low expression of ENO3 is negatively related to OS in HCC patients. Moreover, ENO3 represses the proliferative, migratory, and invasive capacities of HCC cells *in vitro* and *in vivo*. Further research discovers that ENO3 suppresses EMT and inhibits the progression of HCC by modulating the Wnt/β-catenin signaling pathway, which may become an underlying therapeutic target for HCC.

## Materials and Methods

### Database Retrieval for The Analysis of Gene Expression and Survival

The mRNA expression data of the HCC suffers from Japan was acquired from public ICGC datasets, which comprises 177 normal specimens and 212 tumor specimens. RNA expression data of HCC patients were acquired from the Cancer Genomics Brower of the UCSC. In total, 374 tumor specimens and 50 normal specimens were selected in the TCGA-LIHC cohort. Patients without survival information were excluded. The Survival R package was performed to determine the optimal cut-point, and the two groups were classified, including the low- and high-expression groups. K–M survival analysis between low- and high-expression groups was further completed via the Survival R package.

### Human HCC Tissue Specimens

An overall 62 pairs of mankind HCC tissue and paired normal hepatic tissue were randomly collected from HCC suffers who were histopathologically examined and without radiation treatment or chemical treatment prior to resection between June 2019 and August 2020. The entire sufferers received surgeries at the First Affiliated Hospital of Zhengzhou University, China. The research protocols were accepted by the Ethics Committee of the First Affiliated Hospital of Zhengzhou University, China.

### Cell Lines and Cell Cultivation

Mankind liver cancer cell lines (SMMC7721, Huh7, HepG2, MHCC97H) were available in Henan Organ Transplantation Research Center. The cells were cultivated in DMEM (Gibco, United States) added with 1% antibiotics and 10% FBS (Gibco, United States). The culture incubator was sterilized at 37°C with 5% CO2.

### Quantitative Real-Time PCR Assay (qRT-PCR)

The overall RNA of HCC cells or clinical specimens was extracted via Trizol (Invitrogen, United States) and then was reversed to cDNA via HiScript® III first Strand cDNA Synthetic Kit (Vazyme, China). The 2x SYBR Green qPCR Master Mix (Bimake, United States) was utilized for the qRT-PCR assay. Relative quantification of mRNA levels was computed as fold change by the standard formula, 2^-△△CT^= (CT_target_−CT_GAPDH_) specimen− (CT_target_−CT_GAPDH_) control. The sequence of the primers for the qRT-PCR are presented below:

ENO3 sense: 5′-TCT​GGG​GAG​ACT​GAG​GAC​AC-3’; and antisense: 5′-GCC​TTC​GGG​TTA​CGG​AAC​TT-3;

GAPDH sense: 5′-TCT​GGG​GAG​ACT​GAG​GAC​AC-3’; and antisense: 5′-GCC​TTC​GGG​TTA​CGG​AAC​TT-3;

### Nuclear Protein Extraction

The nuclear and cytoplasmic proteins of cells were extracted by the Nucleus and Cytoplasm Abstraction Kit (Solarbio, China) following the supplier’s manual. The entire specimens were reserved at −80°C for the assays later on.

### Western Blot Assay

Cells or tissues were lysed by RIPA buffer added with 1 mM PMSF (Solarbio, China) and Protease Suppressor Cocktail (Thermo Fisher Scientific, United States) and cultivated on ices for 0.5 h. The protein concentrations were identified via the BCA protein analysis kit (Solarbio, China). The specimens were subjected to separation by 10% SDS-PAGE and moved to a PVDF film. Then, the treated films were subjected to 5% nonfat milk in TBST for 120 min under RT and immunoblotted with the specific primary antibody under 4°C nightlong. The films were subsequently cleaned with TBST and cultivated with alkaline phosphatase-conjugated second antibodies for 60 min under RT. Immunoblotting was observed via the Odyssey® Dlx Imaging System. ImageJ software was utilized to analyze the densitometric. The antibodies are presented in Supplementary Table S1.

### Generation of Stable ENO3 Overexpression Cell Lines

Overexpression of ENO3 was performed by pLenti-CMV-ENO3-GFP-Puro plasmid (ENO3). Plenti-CMV-GFP-Puro plasmids were utilized as control (Vector). Plasmids were bought from Public Protein/Plasmid Library (Public Protein/Plasmid Library, China), and the entire constructs were verified *via* sequencing. Briefly, MHCC97H and HepG2 cells were planted in a 6-well plate overnight and then transfected with pcDNA3.1/ENO3 plasmid and pcDNA3.1/Vector plasmid using LipoMax reagent (SUDGEN, China) as per the supplier’s specification. Subsequently, 24 h after transfected, cells were subjected to selection via 1 μg/ml puromycin (Invitrogen, United States) for 14 days. The steady colony formations were screened and kept in 1 μg/ml puromycin.

### Knockdown of ENO3 by siRNA Transduction

ENO3-siRNA and control siRNA (NC) were purchased from Hanbio (China) for ENO3 knockdown experiments. MHCC97H and HepG2 cells were inoculated in a 6-well plate nightlong and afterward transfected with siRNA (final concentration 50 nM) mediated by Lipofectamine™ RNAiMAX reagents (Invitrogen, United States). After 48 h transfection, the cultivation intermediary was discarded, and cells were harvested for the following experiments. SiRNA infection referred to the supplier’s specification. The sequences of ENO3-siRNA and negative control (NC) siRNA were as follows:

ENO3-siRNA-1, sense: 5′- CCA​ACA​UCC​UGG​AGA​ACA​ATT - 3′; and antisense: 5′ - UUG​UUC​UCC​AGG​AUG​UUG​GTT -3′; ENO3-siRNA-2, sense: 5′- CCA​AAU​ACA​ACC​AAC​UCA​UTT - 3′; and antisense:

5′- AUG​AGU​UGG​UUG​UAU​UUG​GTT -3′; negative control (NC), sense: 5′- GUG​GAU​AUU​GUU​GCC​AUC​ATT -3′; and antisense:

5′- UGA​UGG​CAA​CAA​UAU​CCA​CTT -3′.

### Cell Proliferation Analysis

Transfected MHCC97H and HepG2 cells were inoculated in 96-well plates at 1 × 10^3^ cells per well and were examined on days 1, 2, 3, 4, and 5 after transfection. Cellular activities were assessed via Cell Counting kit-8 analysis (Solarbio, China). The original intermediary in every well was substituted by the intermediary with 10% CCK-8 reagent at the scheduled time points. After incubation under 37°C for 120 min, the absorption of samples was identified at 450 nm via a microplate reading device.

### Cell Colony Forming Analysis

Transfected MHCC97H and HepG2 cells were planted in 6-well plates (1,000 cells per well) and cultivated for approximately 14 days. Replacing cultivation intermediary was performed every 3–4 days. Then, the cells were subjected to fixation with 4% PFA and stained *via* 0.1% CVSS for 15 min. After washing with PBS, the colony formations were shot and counted.

### Wound-Healing Assays

The transfected MHCC97H and HepG2 cells were planted into 6-well plates. When they displayed 90% confluence, a standard 200 μL pipet was afterward used to create linear wounds after washing 2 times with PBS. The cells were cultivated with the intermediary without FBS for an additional 48 h. The ranges of wounds were monitored and imaged by microscopy.

### Cell Migration and Invasion Assays

Transfected MHCC97H and HepG2 cells were seeded into the top chambers of Corning Incorporated Cell Culture Inserts with a polycarbonate filtration film (8 μm aperture size, 6.5 mm diameter). For cell migratory ability, 2 × 10^5^ cells in 100 μL of intermediary without FBS were inoculated in the upper chamber without Matrigel (BD, United States). For cell invasive ability, 2 × 10^5^ cells in 100 μL of intermediary without FBS were inoculated in the upper chamber that was precoated with Matrigel. The bottom chamber was prepared with 800 μL DMEM intermediary (added with 20% FBS). After 48 h of incubation under 37°C within a moist environment with 5% carbon dioxide, the non-migrated or non-invaded cells adhering to the upper chamber were removed discreetly, whereas migrated or invaded cells adhering to the lower chamber were subjected to 4% PFA for 15 min and dyed by 0.1% CVSS for 15 min.

### Xenograft Assay

The animal studies were approved by the Ethics Committee of the First Affiliated Hospital of Zhengzhou University, China. 5 weeks old female BALB/C nude mice were bought from Beijing Vital River Laboratory Animal Technology (China) for evaluating the proliferative ability *in vivo.* 5 × 10^6^ MHCC97H cells stably overexpression ENO3, subjected to suspension in 100 μL PBS, were introduced into the right dorsal flank of the animals via subcutaneous implantation. The cancer volume along with bodyweight was identified every other day, and average cancer volume was computed using the equation: volume= (length × width^2^)/2. The entire animals were euthanized and imaged after 3 weeks and cancer tissue was stripped and measured discreetly.

### Lung Metastasis Models

To evaluate the tumor metastasis *in vivo*, 3 × 10^6^ MHCC97H cells stably overexpression ENO3, suspended in 100 μL PBS were injected into animals via the tail vein, and the entire animals were euthanized posterior to 8 weeks. The pulmonary tissue was subjected to dissection discreetly and photographed. The lung was dyed *via* H&E. The entire pulmonary tissue of all mice was sliced, and the pulmonary metastasis nodules were calculated in HPF using a microscopic device.

### Histology and IHC Assay

The clinical samples and pulmonary tissue acquired from the animals were subjected to fixation in 4.0% PFA nightlong at RT. Subsequently, the tissue was fixed in paraffin and sectioned into slices (5 μm). Afterward, the histology section of lung tissue was stained with H&E and observed via an optical microscopic device. For IHC analysis, after deparaffinization and hydration, the section was blocked of endogenic peroxidase via 3% H₂O₂ and pre-treated with heat in EDTA (PH 8.0) by a micro-wave stove for 300 s. After that, the primary antibody and second antibody (37°C, 0.5 h) were cultivated. The slice was dyed via DAB and subjected to counterstain via hematoxylin. The level of immune staining was scored respectively by 2 separate researchers not informed of the histopathology characteristics and sufferer information. The scoring was decided via the combination of the level of positively-dyed oncocytes and the dyeing intensity.

### Small Molecule

CHIR-99021 was purchased from MedChemExpress (United States). The transfected MHCC97H and HepG2 cells were treated with 2 μmol/ml CHIR99021 to reactivate Wnt/β-catenin signal as per the supplier’s specification.

### Statistics Analysis

Data were expressed as mean ± SD from three independently performed assays. The diversity between the experiment group and the controls was compared via the Student’s t-test or one-way ANOVA. The survival rate was computed via the K-M approach, and the difference in survival was assayed *via* a log-rank test. The diversity was thought to have significance at *p < 0.05, **p < 0.01, ***, p < 0.001 vs. Vector group or NC group. The results were assayed with GraphPad Prism 8.0.

## Results

### ENO3 Is Downregulated in HCC Tissue and Associated With the Prognosis of Patients

**FIGURE 1:**
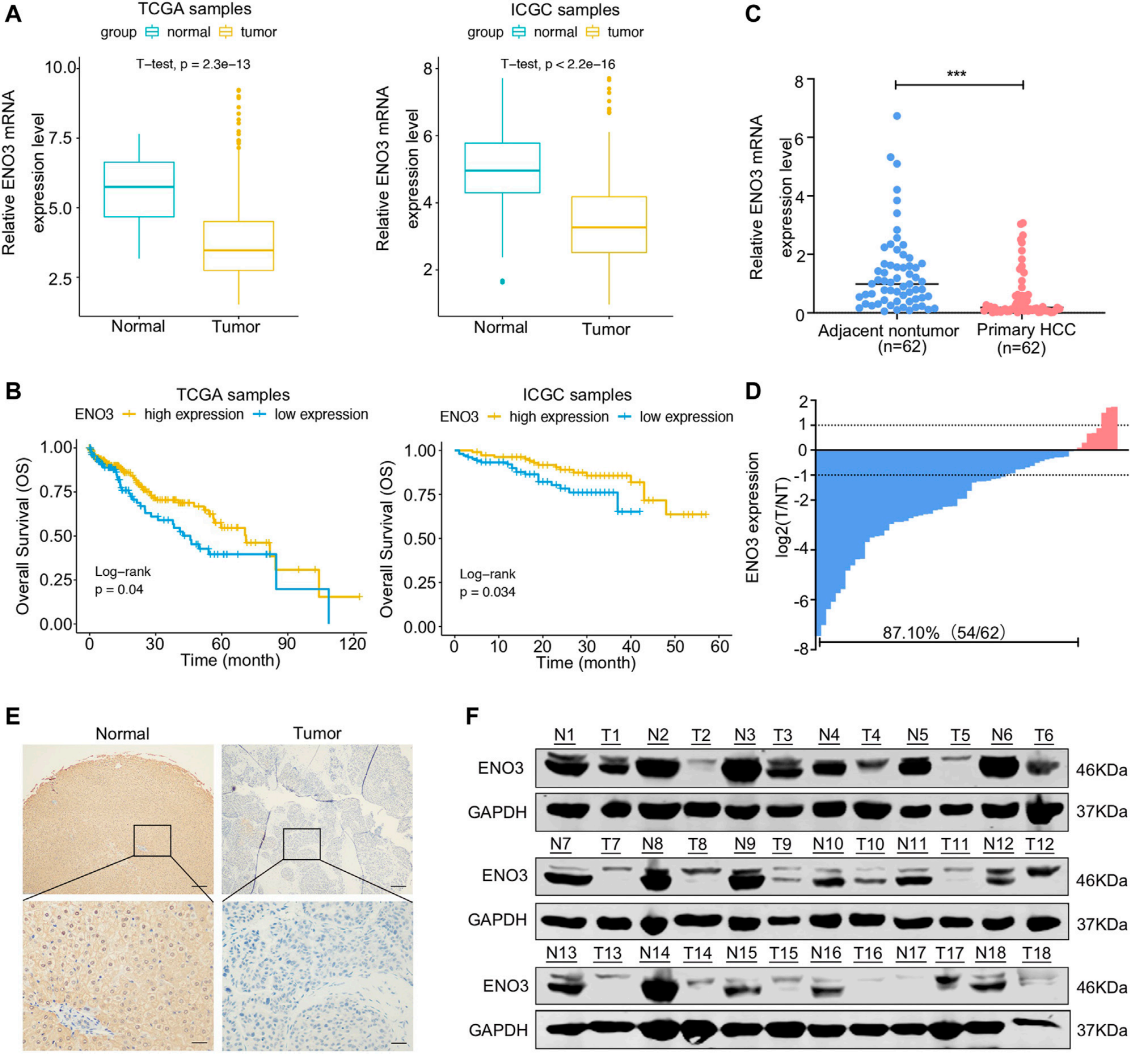
ENO3 is downregulated in HCC tissue and associated with the prognosis of patients. **(A)** The mRNA levels of ENO3 were significantly lower in cancer samples in contrast to that of normal samples as calculated by the TCGA dataset (374 tumor samples and 50 normal samples) and the ICGC dataset (177 normal samples and 212 tumor samples) (*p < 0.001*, Student’s t-test). **(B)** The Survival R package was employed to identify the optimal cut-point, and the two groups were classified, including the low- and high-expression groups. The overall survival rates of HCC patients were compared between the low- and high-ENO3 groups by Kaplan–Meier survival analysis (*p <* 0.05). **(C)** The mRNA levels of ENO3 in 62 primary HCC samples and their neighboring healthy tissue were detected via qRT-PCR. The expression of ENO3 in HCC tissue was lower than that of nontumor tissue (***p < 0.001, Student’s t-test). **(D)** Down-regulation of ENO3 was detected in 87.1% (54/62) of tumor tissue. Data were described as the log2 ratio of the ENO3 mRNA levels in HCC samples (T) in contrast to their matched surrounding NT samples. **(E,F)** Immunohistochemistry and western blot were employed to identify the expression of ENO3 protein in T and NT tissue (*n* = 18, Scale bar, 250 μm above and 100 μm below).

To identify the level of ENO3 in HCC, we firstly assayed the expression of mRNA and prognostic significance of ENO3 in HCC using TCGA and ICGC databases. The analyses of data acquired from these databases demonstrated a remarkable downregulation of ENO3 in the HCC tissue in contrast to normal liver tissue (Figure 1A). In addition, the K-M survival assay of TCGA and ICGC databases displayed that HCC sufferers with higher expression of ENO3 exhibited more prolonged OS compared with sufferers with low expression of ENO3 (Figure 1B). To validate the expression of ENO3 in HCC, total mRNA from 62 HCC tissue and paired neighboring normal hepatic tissue were extracted for qRT-PCR analysis to detect the effect of ENO3 on HCC progression. The expression of ENO3 was notably decreased in HCC tissue (Figure 1C). Furthermore, down-regulation of ENO3 (T/NT ≤ 1) was observed in 87.10% (54/62) of the HCC tissue in contrast to their matched neighboring healthy hepatic tissue (Figure 1D). We also explored the expression of ENO3 in HCC tissue using immunohistochemistry analysis (IHC) (Figure 1E). Finally, we detected ENO3 in 18 HCC tissue and the corresponding neighboring liver tissue by western blot, and it was consistent with this result of qRT-PCR and IHC (Figure 1F). Together, these data strongly suggested that ENO3 was downregulated in HCC, and the low expression was related to undesirable clinical outcomes in the HCC patients, which provided prima facie evidence that ENO3 might be pivotal for HCC.

### ENO3 Suppresses Proliferation of HCC Cells *in Vitro*

**FIGURE 2:**
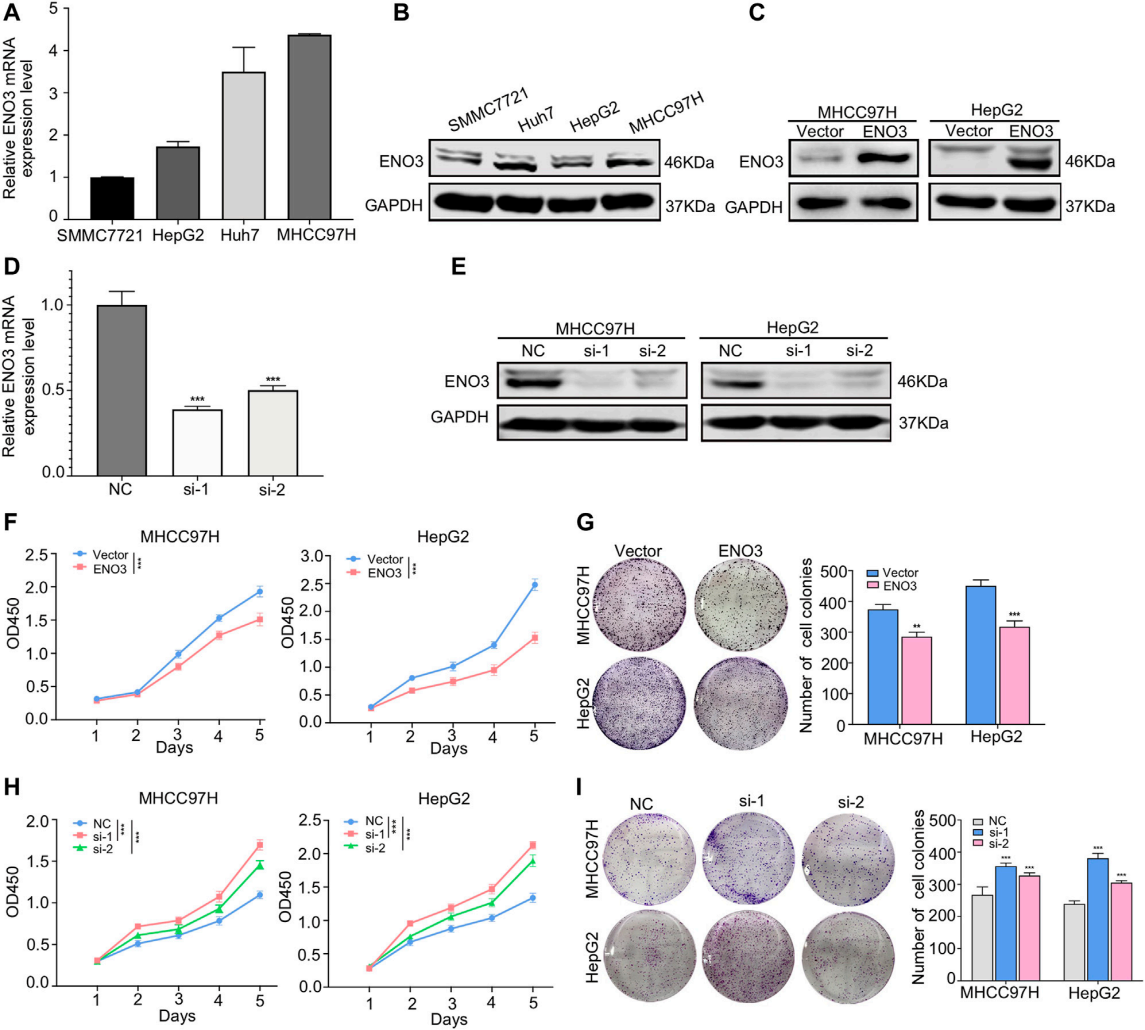
ENO3 suppresses proliferation of HCC cells *in vitro*. **(A,B)** The mRNA and protein levels of ENO3 in Human hepatic carcinoma cells cell lines (SMMC7721, Huh7, HepG2, MHCC97H) were examined by qRT-PCR and western blot. **(C)** Successful ENO3 overexpression was confirmed by and western blot in MHCC97H and HepG2 cells. **(D,E)** The ENO3 knockdown effects were confirmed by qRT-PCR and western blot. GAPDH was utilized as the inner loading control. **(F,G)** Cellular proliferative ability was identified via the CCK-8 analysis with ENO3 overexpression or depletion in MHCC97H and HepG2 cells. **(H,I)** The effect of ENO3 on colony formation in HCC cells. **p < 0.01, ***p < 0.001 vs. Vector group or NC group.

To investigate the potential functions of ENO3 in HCC, we firstly detected the expression of ENO3 in different human HCC cell lines (SMMC7721, Huh7, HepG2, and MHCC97H). QRT-PCR and western blot analysis uncovered that ENO3 expressed differently in the four HCC cell lines (Figures 2A,B). Then we overexpressed ENO3 by using plasmid and knocked it down by two independent small interfering RNAs (siRNAs) both in MHCC97H and HepG2 cells. Successful ENO3 overexpression (Figure 2C) or reduction was confirmed by qRT-PCR and western blot assays (Figures 2D,E). Compared with vector cells, overexpression of ENO3 significantly inhibited cell proliferation (Figure 2F) and produced fewer and smaller colonies in MHCC97H and HepG2 cells (Figure 2G). In contrast, knockdown of ENO3 notably promoted cell proliferation (Figure 2H) and generated more and larger colonies (Figure 2I). Therefore, these results demonstrated that ENO3 could suppress the proliferative ability of HCC cells *in vitro*.

### ENO3 Represses Migration and Invasion of HCC Cells Through Inhibiting EMT *in Vitro*

**FIGURE 3:**
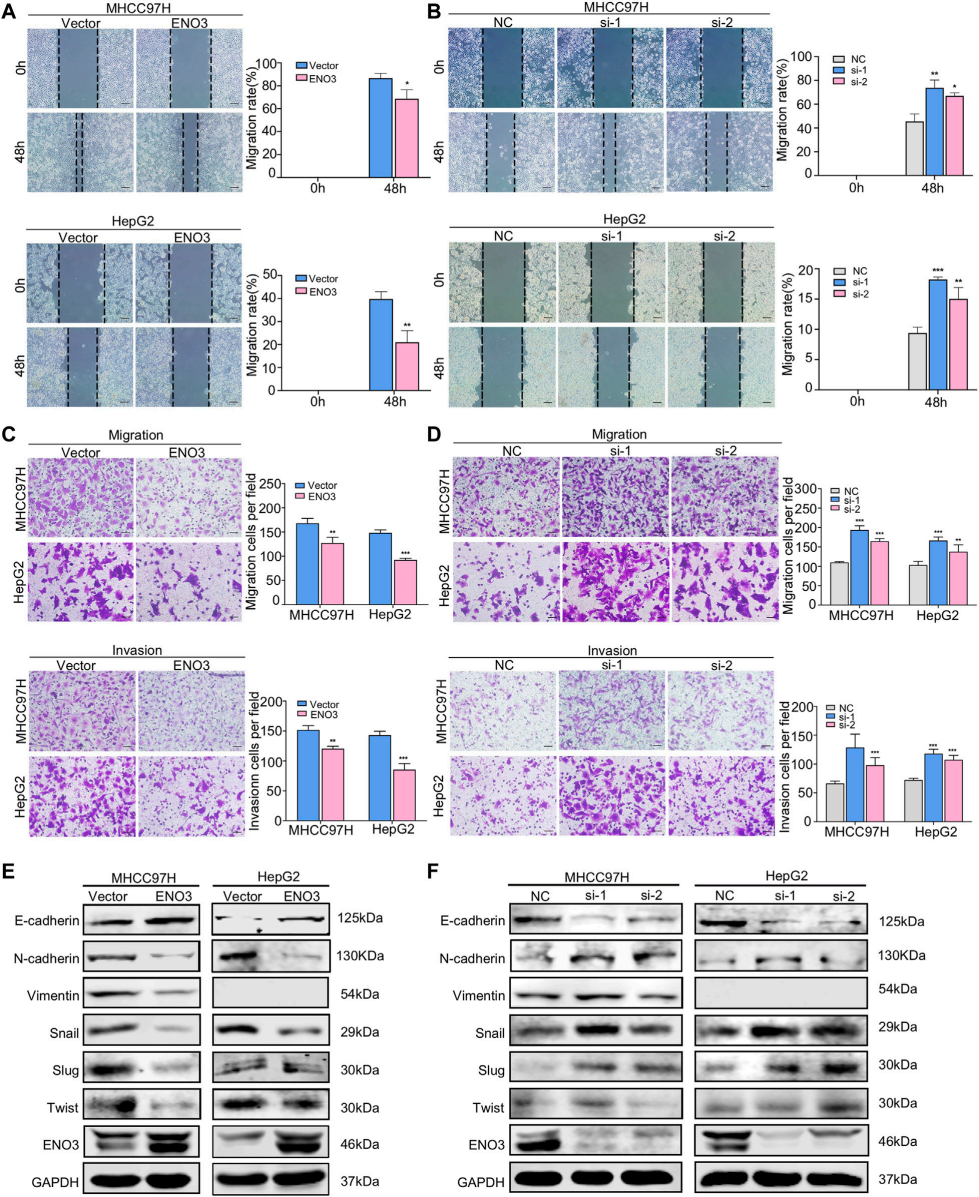
ENO3 represses migration and invasion of HCC cells through inhibiting EMT *in vitro*. **(A,B)** The migration activity of HCC cells was examined *via* wound-healing assay after enforcing or knocking down the expression of ENO3. The wound space was photographed at 0, 48 h (Scale bar, 200 μm). **(C,D)** Transwell assay showed the migration and invasion of HCC cells with ENO3 overexpression or ENO3 deletion (Scale bar, 50 μm). **(E,F)** The EMT-associated proteins, such as E-cadherin, N-cadherin, Vimentin, Snail, Slug, and Twist, were analyzed *via* western blot after overexpression or knocking down ENO3. *p < 0.05, **p < 0.01, ***, p < 0.001 vs. Vector group or NC group.

A previous study reported that ENO3 was involved in the genes related to the metastasis potential of HCC by bioinformatic analysis (Ye et al., 2003), whereas it had not been experimentally demonstrated. To further validate the roles of ENO3 in HCC, wound-healing assays were used to assess the migratory ability of HCC cells. In contrast to the control group, cells with elevated expression of ENO3 displayed a more intensive area of wound closure, while cells with knocked down expression of ENO3 exhibited a more extensive area of wound closure (Figures 3A,B). Furthermore, we investigated the cellular migratory ability (via Transwell chamber analysis, without Matrigel) and invasive ability (cellular migratory events across Transwell chamber coated with Matrigel) in MHCC97H and HepG2 cells. A considerable decrease in cellular migratory and invasive events was identified in the ENO3 overexpressed cells (Figure 3C), and a remarkable increase in cellular migratory and invasive events was determined in the ENO3 depressed cells (Figure 3D), compared with the controls, respectively.

Since it becomes increasingly known that EMT, where cells lose the epithelial identity and obtain mesenchymal characteristics, is significant in tumor propagating potential and essential for cancer cell migration and invasion (Bakir et al., 2020). We speculated ENO3 might be important for the EMT process. To identify such an assumption, we examined the genes associated with EMT and found that overexpression of ENO3 decreased the expression of mesenchymal marker N-cadherin, Vimentin Snail, Slug, and Twist, and increased the expression of epithelial marker E-cadherin (Figure 3E). In contrast, silencing of ENO3 led to adverse effects (Figure 3F). Taken together, the above results firmly demonstrated that ENO3 suppressed migration and invasion of HCC cells by inhibiting EMT *in vitro*.

### ENO3 Suppresses Wnt/β-Catenin Signaling Pathway in HCC Cells

**FIGURE 4:**
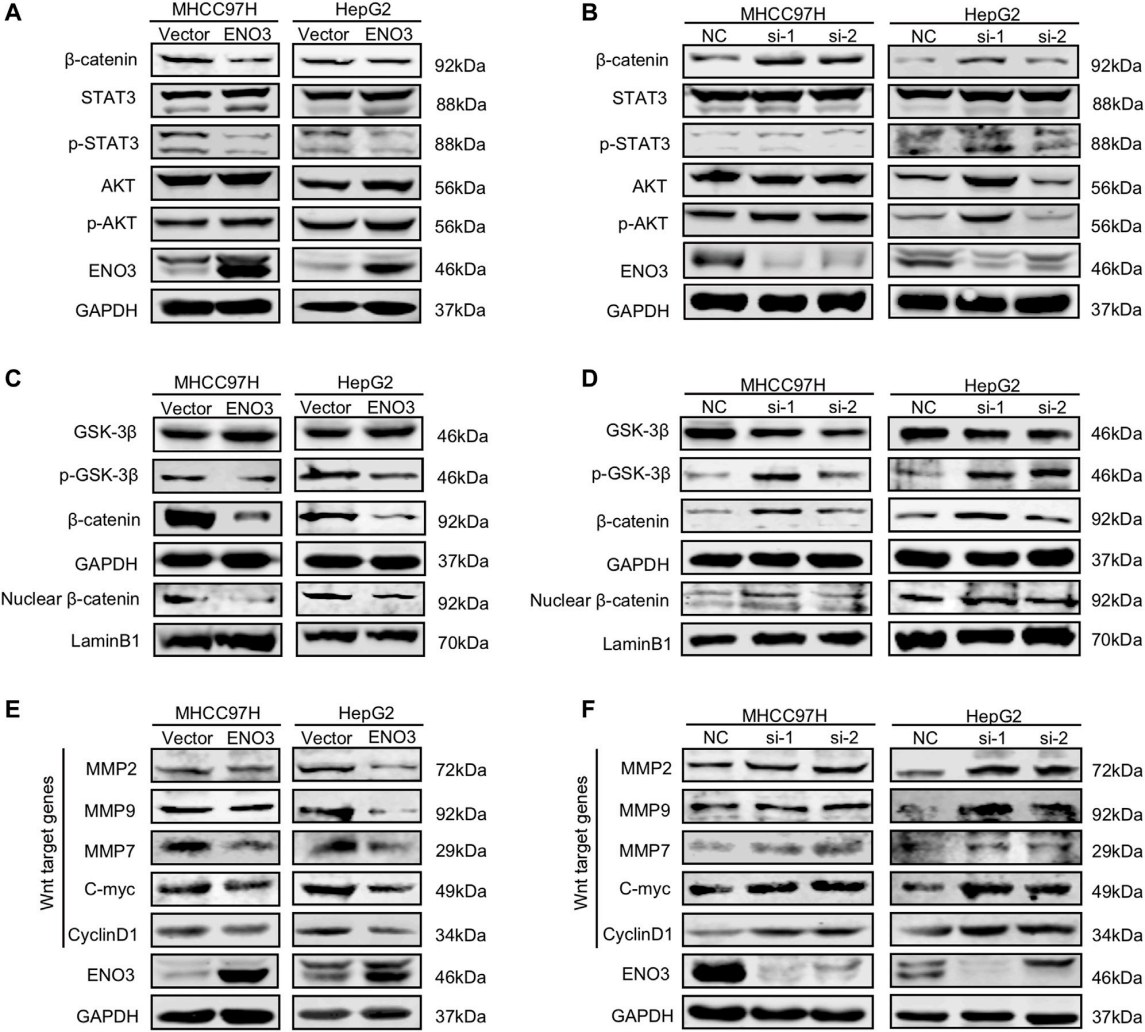
ENO3 suppresses Wnt/β-catenin signaling pathway in HCC cells. **(A,B)** Western blot analysis was performed to determine the protein content of β-catenin, STAT3/p-STAT3, and AKT/p-AKT in MHCC97H and HepG2 cells overexpressed or knocked down ENO3. **(C,D)** GSK-3β/p-GSK-3β, overall β-catenin, and nuclear β-catenin protein content of the cells were analyzed via western blot. GAPDH and Lamin B1 were utilized as the inner loading control, separately. **(E,F)** Western blot was used to determine Wnt targeted genes in MHCC97H and HepG2 cells with ENO3 overexpression or ENO3 knockdown.

For the purpose of investigating the potential molecular mechanism of ENO3-triggered inhibition of oncogenic events in HCC, we detected the involvement of ENO3 in JAK/STAT, PI3K/AKT, and Wnt/β-catenin signaling pathways of which the activities are closely related to the carcinogenesis and progression of HCC (Moeini et al., 2012; Khemlina et al., 2017). ENO3 overexpression in MHCC97H and HepG2 cells notably repressed the expression of β-catenin but failed to influence the expression of AKT/Phospho-AKT, STAT3/Phospho-STAT3 (Figure 4A). Moreover, the expression of β-catenin was elevated while the expression of AKT/Phospho-AKT, STAT3/Phospho-STAT3 still had no change upon ENO3 depletion (Figure 4B). Those outcomes revealed that ENO3 might regulate Wnt/β-catenin signaling transmission in HCC cells in a selective way. GSK-3β-mediated β-catenin phosphonation and degradation was the main approach that modulated β-catenin expression levels, and p-GSK-3β could impede β-catenin degradation (He and Tang, 2020). The accumulated β-catenin then translocated from cytoplasm to nucleus and stimulated the canonical Wnt/β-catenin signaling pathway (Perugorria et al., 2019; He and Tang, 2020). Therefore, we investigated the protein expressions of GSK-3β, p-GSK-3β, overall β-catenin, and nuclear β-catenin. We found that forced expression of ENO3 not only inhibited the expression of p-GSK-3β but also suppressed the level of β-catenin protein both in total and in the nucleus (Figure 4C), while knockdown of ENO3 elevated p-GSK-3β, overall β-catenin and promoted the nuclear transfer (Figure 4D). To further confirm the modulation of β-catenin by ENO3, then we assessed the expression of Wnt/β-catenin signaling pathway downstream targeted genes, including *C-myc*, *CyclinD1*, *MMP2*, *MMP7*, and *MMP9*. Compared to the controls, enhanced expression of ENO3 significantly inhibited the level of C-myc, Cyclin D1, MMP2, MMP7, and MMP9 (Figure 4E), whereas knockdown of ENO3 markedly increased the expression of C-myc, CyclinD1, MMP2, MMP7, and MMP9 (Figure 4F). Altogether, these results implied that ENO3 might suppress carcinogenesis and the malignant phenotype of HCC *via* inhibiting the activation of the Wnt/β-catenin signaling pathway.

### Activating Wnt/β-Catenin Pathway Abolishes the Suppressive Effects of ENO3 on HCC Cells

**FIGURE 5:**
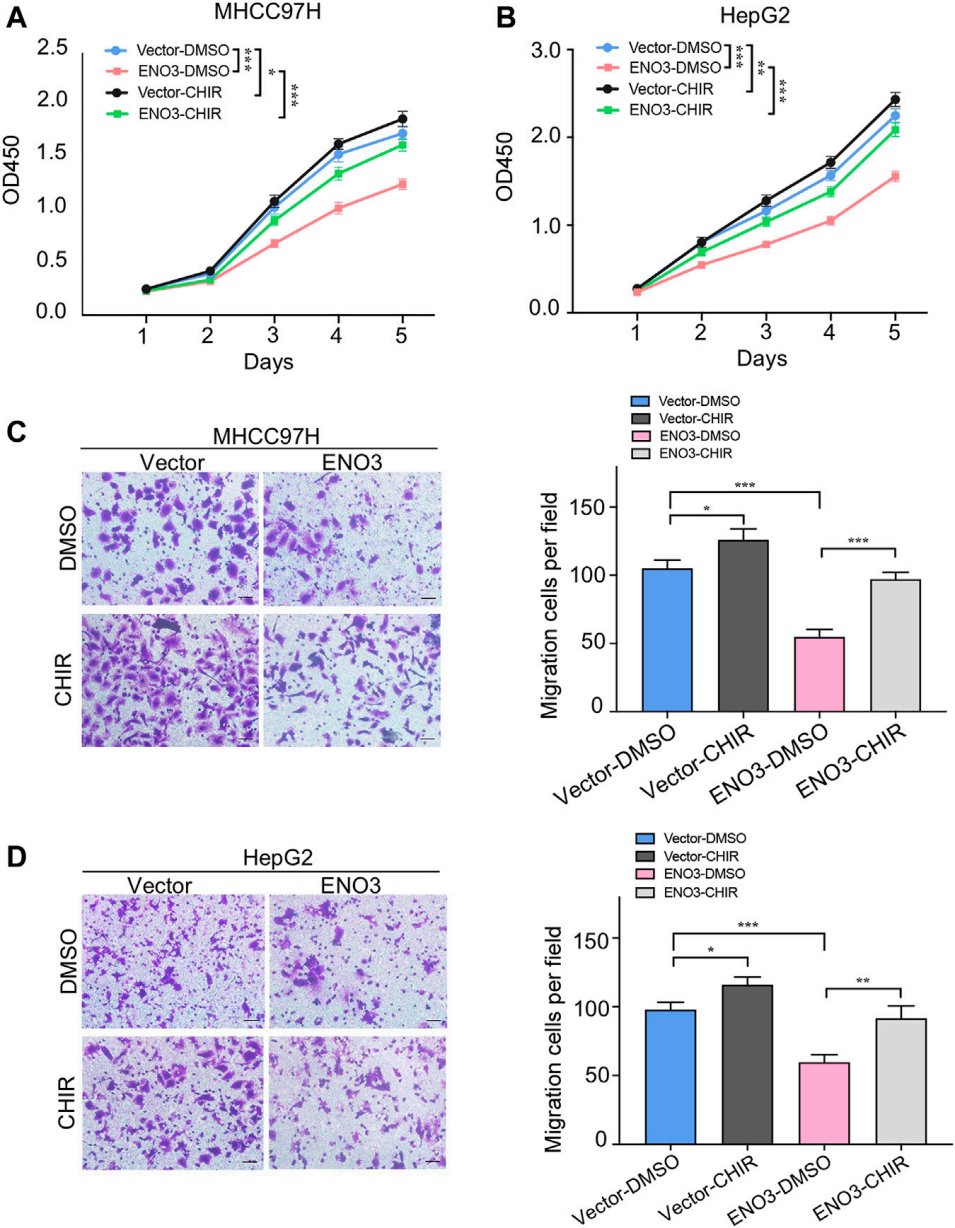
Activating Wnt/β-catenin pathway abolishes the suppressive effects of ENO3 on HCC cells. **(A,B)** The proliferation of MHCC97H and HepG2 cells that overexpressed ENO3 was determined by the CCK-8 assay with CHIR-99021 treatment. **(C,D)** Transwell assay showed the migration of HCC cells with ENO3 overexpression and CHIR-99021 treatment (Scale bar, 50 μm). CHIR-99021 concentration: 2 μmol/ml. *p < 0.05, **p < 0.01, ***, p < 0.001 vs. Vector-DMSO group or ENO3-DMSO group.

To future verify the necessity of the Wnt/β-catenin signal in the suppressive effects of ENO3 on HCC, we utilized CHIR-99021, known as a Wnt/β-catenin signal agonist for rescue experiments (Weng et al., 2020). The CCK-8 assays show the inhibited proliferation capacity of MHCC97H and HepG2 cells that overexpressed ENO3 was significantly recovered with treatment of CHIR-99021 (Figures 5A,B). Furthermore, Transwell assays also revealed that cell migration was repressed by overexpression of ENO3, which was weakened by CHIR-99021 treatment (Figures 5C,D). These results indicated that CHIR-99021 could reverse the inhibitory effects caused by ENO3, suggesting ENO3 exerts tumor-suppressor functions via the Wnt/β-catenin pathway.

### ENO3 Inhibits Growth and Metastasis of HCC Cells *in Vivo*

**FIGURE 6:**
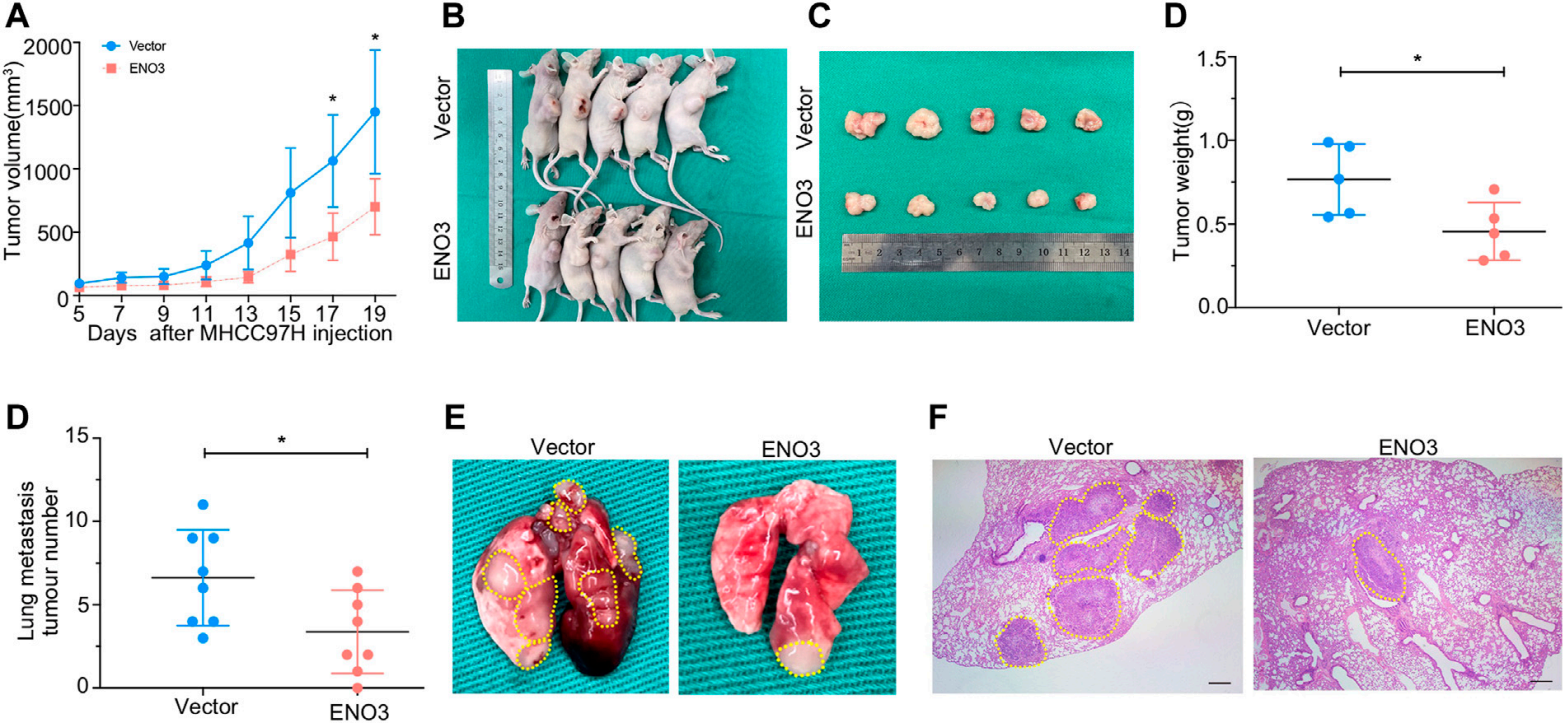
ENO3 inhibits proliferation and metastasis of HCC cells *in vivo*. **(A)** The tumorous growth curve of ENO3 overexpressed cells was contrasted with vector control cells. MHCC97H cells stably overexpressed ENO3 stable transfectants and control vector stable transfectants were introduced into the right flank of the animals via subcutaneous injection, separately (5 × 10^6^ cells every mouse, *n* = 5 for each group). The cancer volume was computed via the formula: volume = (length × width^2^)/2. **(B,C)** Images of BALB/c-nude mice in each group 21 days after subcutaneous injections of ENO3-overexpression or vector in MHCC97H cells. **(D)** Weights of tumor nodules from subcutaneous mouse xenograft model. **(E)** Quantity of lung metastases in the mice posterior to tail vein injections with ENO3 overexpressed MHCC97H cells and control cells (3 × 10^6^ cells every mouse, *n* = 8 for each group). **(F,G)** Typical lungs and H&E images of lung cancers were presented (Scale bar, 250 μm). *p < 0.05.

The effects of ENO3 on proliferation, migration, and invasion were confirmed *in vitro*, we afterward investigated the role of ENO3 in cancer formation *in vivo* by a xenograft mouse model. The MHCC97H cells that stably overexpressed ENO3 were introduced into the right flank of the animals via subcutaneous implantation. Cells overexpressed ENO3 exhibited much lower tumor growth rates than vector cells with a dramatic reduction in the tumor volume (Figure 6A). Tumors were excised 3 weeks after HCC cells inoculation, and their images were photographed (Figures 6B,C). The tumor weight of the animals within the ENO3 group was significantly lighter compared with the vector group (Figure 6D). We also verify the suppressive role of ENO3 in distant seeding of HCC cells by using a tail vein injection model. Eight weeks posterior to injections, the number of metastatic nodules was notably less in mice injected with overexpression of ENO3 group in contrast to the control group (Figures 6E–G). Conform to the *in vitro* study, these results demonstrated that ENO3 repressed proliferation and metastasis of HCC cells *in vivo*.

## Discussion

Up till now, the effects of ENO3 on tumors remain controversial, and its effects might be different depending on a variety of cell types. For example, previous researches reported that ENO3 was highly expressed in *STK11* mutant lung cancer, colorectal cancer, and its ectopic expression correlated with a worse outcome of patients by bioinformatic analysis (Park et al., 2019; Zhu et al., 2021). However, the results were converse in pancreatic carcinoma and HCC (Liu et al., 2019; Tan et al., 2020). Based on results derived from the TCGA database, ICGC database, and clinical samples, we found that ENO3 was notably downregulated in human HCC samples in contrast to the neighboring normal tissue. Furthermore, the sufferers with low expression of ENO3 exhibited shorter OS in contrast to those with ENO3 high expression. These findings indicated ENO3 might act as a significant role in the development of HCC. For the purpose of exploring the function of ENO3 in HCC, we stably overexpressed ENO3 both in MHCC97H and HepG2 cells. The results showed that overexpression of ENO3 significantly inhibited cellular proliferative, migratory, and invasive abilities, as well as cellular colony forming *in vitro*, whereas silencing of ENO3 in MHCC97H and HepG2 cells led to the opposite effect. We also demonstrated the growth and metastasis suppressor role of ENO3 in different primary HCC mouse models. Therefore, we disclosed a suppressor function of ENO3 in HCC in this work.

EMT process was positively correlated with variant cancer functions, such as cancer onset, malignance development, cancer stemness, metastasis, drug resistance, and metabolic reprogramming (Tiwari et al., 2012; Cho et al., 2020). E-cadherin, N-cadherin, and Vimentin were the primary biomarkers of EMT (Diepenbruck and Christofori, 2016; Aiello and Kang, 2019). In the present research, we discovered that high expression of ENO3 was related to the upregulated expression of E-cadherin and downregulation of N-cadherin, Vimentin. Consistently, the result was verified by the knockdown of ENO3. After screening the major EMT-activating TFs, including Snail, Slug, and Twist (Wan et al., 2020; Yu et al., 2021), we also confirmed that all of them exhibited mutually exclusive expression patterns in HCC cells with modulated ENO3 expression. These findings suggested that ENO3 might act as a suppressor by inhibiting EMT in HCC cells.

Signaling pathways that are associated with embryogenesis usually become overactive during tumorigenesis (Dimri and Satyanarayana, 2020). Accumulating evidence revealed that many signaling pathways, like Wnt/β-catenin, JAK/STAT, and PI3K/AKT signaling pathways modulated the EMT process and facilitated various cellular abilities like proliferative ability, cellular cycle development, invasiveness, and chemotherapy resistance in various tumors (Moeini et al., 2012; Banerjee and Resat, 2016; Khemlina et al., 2017; Song et al., 2019). To investigate the potential mechanism for ENO3 inhibited proliferation and EMT of HCC, we explored the three signaling pathways. The outcomes herein revealed overexpression ENO3 remarkably reduced the protein level of β-catenin, and knockdown of ENO3 significantly reinforced the expression of β-catenin. The JAK/STAT and PI3K/AKT signaling pathways appear not to be influenced by the ENO3 states within HCC cells, as phosphorylated AKT and phosphorylated STAT3 levels failed to vary when ENO3 was upregulated or downregulated. Previous studies revealed that EMT is commonly overactive in HCC and through its role in GSK-3β phosphonation and downstream impact on β-catenin (Galluzzi et al., 2019; Duda et al., 2020). We verified that ENO3 inhibited the expression of p-GSK-3β, which rescued β-catenin from being phosphorylated and degraded. The cytoplasm-to-nucleus transfer of β-catenin is critical in the transduction of the Wnt/β-catenin cascades. Herein, we discovered that overexpressed ENO3 reduced the nuclear cumulation of β-catenin, whereas ENO3 deletion elevated the nuclear translocation of β-catenin. Afterward, the cumulated β-catenin within the nucleus facilitated the expression of the downstream Wnt-response genes, triggering uncontrollable cell events. Amongst these Wnt-response genes, *C-myc* and *Cyclin D1* are associated with the cellular proliferative ability (Ashihara et al., 2015), and *MMP2, MMP9, MMP7* are related to cell aggressiveness (Pittayapruek et al., 2016). In the present paper, we validated that ENO3 possessed modulatory effects on these genes. Forced ENO3 expression reduced the expression of C-myc, Cyclin D1, MMP2, MMP9, and MMP7 while silencing ENO3 resulted in the opposite effects in HCC cells. Moreover, the small molecules CHIR-99021, working as an agonist of the Wnt/β-catenin pathway, reactivated the proliferation and migration ability which were repressed by the overexpressed ENO3 of HCC cells. Based on these results, we concluded that ENO3 inhibited the growth and metastatic ability of HCC cells through the Wnt/β-catenin signaling pathway.

Metabolic reprogramming, known as the Warburg effect, is characterized by enhanced glycolytic activity and lactate fermentation in cancer (Warburg, 1956; Li et al., 2021). Tumor cells rewire metabolic pathways to supply energy and increase tolerance to an oxygen-deficient environment (Li et al., 2021). The connections between aerobic glycolysis and EMT are not well-understood. Accumulated evidence indicated aberrant expression of glycolytic enzymes not only involve in the metabolic reprogramming but also play significant roles in EMT (Hua et al., 2020). Specifically, Phosphoglucose isomerase (PGI), which transformed glucose-6-phosphate into fructose- 6-phosphate in the second step of glycolysis, could promote EMT by frustrating miR-200 and increasing ZEB1/2 in breast cancer cells (Yang et al., 2011). Pyruvate kinase (PK) catalyzed the conversion of phosphoenolpyruvate and ADP to ATP and pyruvate in the last step of glycolysis (Georgakopoulos-Soares et al., 2020). PKM2, a segment variant of PK, inhibited E-cadherin transcription via interacting with TGFβ-induced factor homeobox two and has been proved to function during EMT in human colorectal cancer cells (Tamada et al., 2012). Furthermore, the translocation of PKM2 into the nucleus could elevate β-catenin accumulation, afterward causing the expression of C-Myc (David et al., 2010; Yang et al., 2011). Interestingly, the activation of Wnt/β-catenin signaling has been demonstrated to augment glycolysis *via* C-Myc in tumors (David et al., 2010; Jiang et al., 2021). Hence, there may be a positive feedback loop between C-Myc and PKM2 to drive glycolysis and EMT. Our findings showed that ENO3 inhibited C-Myc and EMT simultaneously by repressing the Wnt/β-catenin signaling pathway, which strengthened the understanding of metabolic regulatory protein. The specific mechanism of ENO3 in regulating glycolysis and EMT remains to be further investigated.

Despite great advances in diagnostic methods, most HCC patients were still diagnosed in advanced stages, when therapies are mostly limited (Huang et al., 2020). Moreover, anti-cancer agents often show less efficacy due to the difficulty of reaching the tumor site and exerting their anti-tumor activity (Deldar Abad Paskeh et al., 2021). Since Wnt/β-catenin signaling can enhance proliferation and migration rates and drug resistance of HCC cells, thus, ENO3 may serve as a Wnt/β-catenin axis inhibitor and a specific therapeutic target for the treatment of HCC.

**FIGURE 7:**
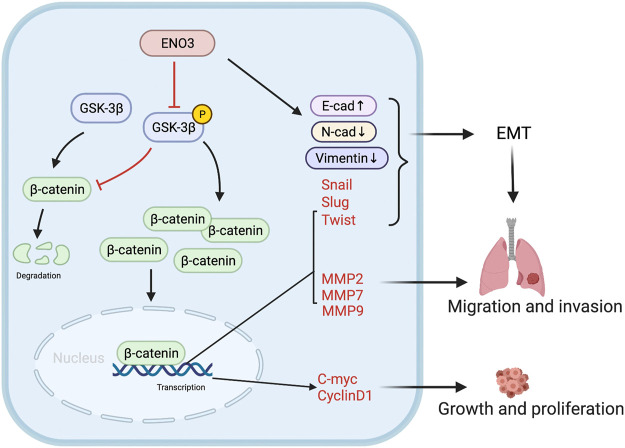
Schematic diagram. The schematic illustration displays that ENO3 down-regulates p-GSK-3β, triggering the β-catenin cumulation in the nucleus and afterward stimulating the EMT and Wnt targeted genes, which facilitates the tumorigenesis and metastasis.

Taken together, our work firstly disclosed that ENO3 serves as a pivotal suppressor in HCC proliferation and metastasis. In addition, we firstly revealed that ENO3 suppresses the EMT process by modulating the Wnt/β-catenin signaling transmission cascades (Figure 7). Holistically, these discoveries provided significant insights into the HCC proliferation and metastasis and put forward ENO3 as a diagnostic and therapeutic target for HCC patients.

## Data Availability

The original contributions presented in the study are included in the article/Supplementary Material, further inquiries can be directed to the corresponding author.

## Abbreviation

CHIR, CHIR99021; CVSS, Crystal Violet Staining Solution; DAB, Diaminobenzidine; DMEM, Dulbecco’s Modified Eagle’s Medium; DMSO, Dimethyl sulfoxide; EDTA, ethylene diamine tetraacetic acid; EMT, epithelial-mesenchymal transition; ENO3, β-enolase; FBS, fetal bovine serum; H&E, hematoxylin and eosin; HBV or HCV, hepatitis B or C virus; HCC, hepatocellular carcinoma; HPF, high-power fields; ICGC, International Cancer Genome Consortium; IHC, Immunohistochemistry; K-M, Kaplan–Meier; LIHC, Liver Hepatocellular Carcinoma; NC, negative control; NT, non-tumor; OS, overall survival; PC, polycarbonate; PFA, paraformaldehyde; PMSF, phenylmethylsulphonyl fluoride; RT, room temperature; siRNAs, small interfering RNAs; TBST, Tris-buffered saline/0.2% Tween-20; TCGA, The Cancer Genome Atlas; TFs, transcription factors; TFs, transcription factors; UCSC, University of California, Santa Gruz.
