# Supplementary material for: ENO3 Inhibits Growth and Metastasis of Hepatocellular Carcinoma via Wnt/β-Catenin Signaling Pathway
Source: Front Cell Dev Biol. 2021 Dec 23;9:797102. doi: 10.3389/fcell.2021.797102 (PMC8733707; doi:10.3389/fcell.2021.797102)
Supplement: Supplementary file 1 [file Table1.docx]

| **Supplementary Table 1 Antibodies for western blot.** | | | | |
| --- | --- | --- | --- | --- |
| **Antibody** | **Concentration** | **Specificity** | **Catalog number** | **Company** |
| ENO3 | 1:1000 | Rabbit Polyclonal | 55234-1-AP | Proteintech |
| GAPDH | 1:5000 | Mouse Monoclonal | 60004-1-Ig | Proteintech |
| E-cadherin | 1:5000 | Rabbit Polyclonal | 20874-1-AP | Proteintech |
| Vimentin | 1:5000 | Mouse Monoclonal | 60330-1-Ig | Proteintech |
| N-Cadherin | 1:2000 | Rabbit Polyclonal | 22018-1-AP | Proteintech |
| GSK-3β | 1:1000 | Rabbit Polyclonal | 22104-1-AP | Proteintech |
| p-GSK-3β | 1:1000 | Mouse Monoclonal | 67558-1-Ig | Proteintech |
| MMP2 | 1:1000 | Rabbit Polyclonal | 10373-2-AP | Proteintech |
| MMP7 | 1:1000 | Rabbit Polyclonal | 10374-2-AP | Proteintech |
| MMP9 | 1:1000 | Rabbit Polyclonal | 10375-2-AP | Proteintech |
| Lamin B1 | 1:2000 | Rabbit Polyclonal | 12987-1-AP | Proteintech |
| Snail | 1:1000 | Rabbit Polyclonal | 13099-1-AP | Proteintech |
| Slug | 1:500 | Mouse Monoclonal | sc-166476 | Santa Cruz |
| Twist | 1:500 | Mouse Monoclonal | sc-81417 | Santa Cruz |
| AKT | 1:1000 | Rabbit Polyclonal | WL0003b | Wanleibio |
| p-AKT | 1:1000 | Rabbit Polyclonal | WLP001a | Wanleibio |
| β-catenin | 1:1000 | Rabbit Polyclonal | WL0962a | Wanleibio |
| C-myc | 1:1000 | Rabbit Polyclonal | WL01781 | Wanleibio |
| CyclinD1 | 1:1000 | Rabbit Polyclonal | WL01435a | Wanleibio |
| STAT3 | 1:1000 | Rabbit Monoclonal | 30835S | Cell Signaling Technology |
| p-STAT3 | 1:1000 | Rabbit Monoclonal | 9145S | Cell Signaling Technology |
